# Pitfalls of onco-metabolomics: impact of sample integrity on metabolomic investigations in more than 4500 human serum samples from ten different cohorts

**DOI:** 10.3389/fmolb.2026.1765747

**Published:** 2026-03-31

**Authors:** Michael Ladurner, Selina Strathmeyer, Tobias Ameismeier, Helmut Klocker, Eberhard Steiner, Gerhard Aigner, Martin Puhr, Tina Böld, Diana Drettwan, Franziska Sommermeyer, Iris E. Eder

**Affiliations:** 1 Department of Urology, Medical University of Innsbruck, Innsbruck, Austria; 2 lifespin GmbH, Regensburg, Germany; 3 Stiftung PATH – Patients’ Tumor Bank of Hope, Munich, Germany

**Keywords:** biobank, breast cancer, human serum, metabolomics, NMR spectroscopy, prostate cancer, sample integrity, pre-analytical/analytical variations

## Abstract

**Introduction:**

Metabolomics such as nuclear magnetic resonance spectroscopy or mass spectrometry (MS) in different body fluids are considered potentially useful diagnostic techniques for various diseases including cancer. One of the most important prerequisites of metabolomics is a high sample quality, for which reason explicit care must be taken during pre-analytical/analytical handling.

**Methods:**

In the present study, we investigated the influence of pre-processing (PPT), and pre-centrifugation time (PCT), sample storage time (SST), and sample texture on NMR-based metabolite levels in 4,658 long-term and short-term stored retrospectively and prospectively collected serum samples from breast and prostate cancer patients as well as from healthy men.

**Results:**

We found that the majority of the metabolites were highly stable with regard to variations in PCT, PPT, or SST. PCT and PPT significantly affected the concentrations of only a few individual metabolites, including ascorbic acid, asparagine, glucose, glutamic acid, glutamine, lactate, phenylalanine, pyruvic acid, and serine, indicating that pre-analytical protocol variations need to be considered for the quantitative analysis of metabolites. Notably, the glucose:lactate and glutamine:glutamic acid ratios were found to be suitable to assess sample quality in case of high PCT or PPT. Importantly, the highest sample quality was detected in prospectively collected serum samples with strict protocol adherence and a total PPT of only 1.2 h. Specific care must also be taken with the analysis of lipemic samples, in which strong variations in the concentrations of lipid metabolites, albumin, and valine were observed.

**Discussion:**

In summary, our data show that the majority of metabolites are mostly stable with regard to variations in pre-analytical processing, indicating that retrospective biobank samples are suitable for metabolomics studies. However, individual metabolites are strongly dependent on PCT and PPT, suggesting that a short PPT may be mandatory for clinical diagnosis, depending on the individual metabolite to be measured.

## Introduction

Mass spectrometry (MS) and nuclear magnetic resonance (NMR) spectroscopy have emerged as commonly used analytical techniques for metabolomics. Characteristic metabolomic profiles have been associated with a variety of diseases such as chronic kidney disease (Hunter et al., 2021), diabetes and cardiovascular disease (Fung et al., 2023), Parkinson`s disease (Li et al., 2022), and various types of cancer such as breast (BCa) (Vignoli et al., 2021), and prostate cancer (PCa) (Krishnan et al., 2023). In particular NMR spectroscopy has great potential as a valid clinical diagnostic method, because it is a highly reproducible and quantitative method, which can be used to analyze various body fluids such as serum, plasma, and urine without any complicated or time-consuming pre-processing (Zniber et al., 2024).

Irrespective of the method used, metabolite quantification is largely influenced by the sample integrity. For the measurement of metabolites in blood/serum samples it has been shown previously that care must be taken during pre-analytical and analytical handling, including blood withdrawal, the type of collection tubes, clotting conditions and durations, temperature and time during transport and processing, storage conditions, and the number of freeze-and-thaw cycles before measurement (Zheng et al., 2021; Ghini et al., 2021; Ghini et al., 2022; Ghini et al., 2019; Salciccia et al., 2021). To enable the comparability of study results from different laboratories, a pre-analytical sample preparation standard, ISO 23118:2021, which defines the most important pre-analytical handling points which may interfere with the analysis, has been set up to minimize technical inter-sample variability (Ghini et al., 2019; Gonzalez-Dominguez et al., 2020).

In the present study, we have addressed several pre-analytical issues, which must be considered when using biobanked serum samples for metabolomics. In particular, we investigated the influence of pre-processing (PPT) and pre-centrifugation times (PCT) as well as sample storage time (SST) in NMR-based metabolomics.

## Materials and methods

### Samples

We used archived serum samples from females with BCa (n = 3,262), and from males (n = 1,396) that were routinely analysed for prostate-specific antigen (PSA) (Table 1). Among these 1,396 men, 773 were healthy or suffered from any benign prostate disease, whereas 623 men had a histologically proven PCa with a mean PSA of 7.72 ng/mL (range: 0.46–252 ng/mL). PCa serum samples were pre-processed and stored in the URO-IBK biobank, which is located at the Department of Urology in Innsbruck, Austria, using two different pre-processing protocols. Clinical characteristics of patients have been published previously (Ladurner et al., 2025). BCa serum samples were pre-processed and stored at seven different hospitals in Germany (PATH, Patients` Tumorbank of Hope), using the same pre-processing protocol. A summary of the different sample cohorts, including the sample numbers, locations, and collection periods is given in Table 1. For this study, we used serum samples from patients with different cancer subtypes, which were however homogeneously distributed across all locations, sample collection dates, and storage periods in order to focus on questions of pre-analytics (Table 2). Written informed consent was obtained from all participants. The use of samples for this study was approved by the Ethics Committee of the BLÄK (Bayerische Landesärztekammer) (approval number: 21071) and the Ethics Committee of the Medical University of Innsbruck (EV 1072/2018 and EV 1329/2021). The URO-PRO cohort serum samples were collected and stored independently in a prospective clinical trial, which was also approved by the Ethics Committee of the Medical University of Innsbruck (EV 1117/2023).

**TABLE 1:** List of serum sample cohorts in this comparative study. Number of samples, collection periods and the main demographical features of the donors are provided.

Biobank name	Cohort name	Collection period, dates	Number of samples, n	Mean age, years (min-max)	Sex (f, m)
URO-IBK	URO-IBK pre2012	2004–2011	593	59.2 (35–79)	m
URO-IBK	URO-IBK 2012+	2012–2021	424	60.8 (40–80)	m
URO-IBK	URO-IBK 2023+	2023–2024	97	64.1 (35–83)	m
URO-PRO	URO-PRO	2023–2025	282	64.2 (35–84)	m
PATH	Center A	2004–2017	1,091	58.9 (19–107)	f
PATH	Center B	2006–2017	337	58 (27–88)	f
PATH	Center C	2006–2016	220	61.5 (25–92)	f
PATH	Center D	2006–2013	239	59.4 (25–87)	f
PATH	Center E	2005–2017	768	57.2 (25–89)	f
PATH	Center F	2005–2017	504	58.9 (23–87)	f
PATH	Center G	2007–2011	103	58.6 (33–85)	f

**TABLE 2:** Distribution of samples over the different cohorts with respect to disease characteristics.

Subgroup	Center A n (%)	Center B n (%)	Center C n (%)	Center D n (%)	Center E n (%)	Center F n (%)	Center G n (%)	URO-IBK n (%)
eBC ER + HER2- G1	133 (38.6)	30 (8.7)	5 (1.4)	1 (0.3)	129 (37.4)	36 (10.4)	11 (3.2)	—
eBC ER + HER2- G2	502 (42.8)	96 (8.2)	72 (6.1)	53 (4.5)	186 (15.9)	224 (19.1)	40 (3.4)	—
eBC ER + HER2- G3	93 (36.2)	34 (13.2)	18 (7)	2 (0.8)	34 (13.2)	60 (23.3)	16 (6.2)	—
eBC HER2+	167 (39.3)	30 (7.1)	61 (14.4)	59 (13.9)	54 (12.7)	35 (8.2)	19 (4.5)	—
eBC TNBC	96 (29.1)	39 (11.8)	51 (15.5)	36 (10.9)	57 (17.3)	35 (10.6)	16 (4.8)	—
pM1 ER + HER2-	19 (35.8)	1 (1.9)	8 (15.1)	6 (11.3)	10 (18.9)	9 (17)	0 (0)	—
pM1 HER2+	3 (16.7)	2 (11.1)	2 (11.1)	1 (5.6)	6 (33.3)	3 (16.7)	1 (5.6)	—
pM1 TNBC	4 (66.7)	0 (0)	1 (16.7)	0 (0)	0 (0)	1 (16.7)	0 (0)	—
ypT0	23 (13.1)	29 (16.6)	1 (0.6)	16 (9.1)	87 (49.7)	19 (10.9)	0 (0)	—
ypT1	27 (8.7)	49 (15.9)	0 (0)	50 (16.2)	139 (45)	44 (14.2)	0 (0)	—
ypT2	18 (13.7)	18 (13.7)	1 (0.8)	14 (10.7)	51 (38.9)	29 (22.1)	0 (0)	—
ypT3	6 (20.7)	6 (20.7)	0 (0)	0 (0)	10 (34.5)	7 (24.1)	0 (0)	—
ypT4	0 (0)	3 (27.3)	0 (0)	1 (9.1)	5 (45.5)	2 (18.2)	0 (0)	—
ISUP GG 1	—	—	—	—	—	—	—	178 (12.8)
ISUP GG 2	—	—	—	—	—	—	—	276 (19.8)
ISUP GG 3	-	—	—	—	—	—	—	97 (6.9)
ISUP GG 4	—	—	—	—	—	—	—	26 (1.9)
ISUP GG 5	—	—	—	—	—	—	—	45 (3.2)
ISUP NA	—	—	—	—	—	—	—	1 (0.072)
Benign prostate	—	—	—	—	—	—	—	773 (55.3)

Abbr: eBC (early breast cancer), ER+ (estrogen receptor positive), ISUP (International Society of Urological Pathology), GG (grade group), G1 (grade 1), G2 (grade 2), G3 (grade 3), HER2+/- (human epidermal growth factor 2 positive/negative), pM1 (pathologically confirmed distant metastasis), TNBC (triple negative breast cancer), ypT (pathological tumor stage after neoadjuvant therapy).

### Sample processing

**FIGURE 1:**
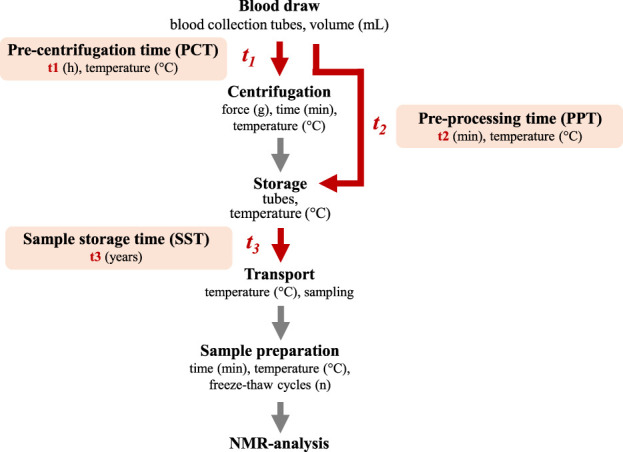
Pre-analytical workflow from sample collection to NMR analysis. Following withdrawal, blood was centrifuged and the serum transferred into a cryo-preservation tube for long-term storage. We defined the time from blood withdrawal until centrifugation as pre-centrifugation time (PCT, t1) and the total time from blood withdrawal until freezing the sample for long term storage as the pre-processing time (PPT, t2). The sample storage time (SST, t3) was defined as the time from freezing the sample until thawing for sample preparation. Times which were investigated in this study were highlighted in red.

The pre-analytical workflow from blood withdrawal to NMR-based metabolite analysis is summarized in Figure 1. In this study, we focused on the time from blood withdrawal until centrifugation of the sample, which was designated the pre-centrifugation time (PCT), the total time from blood withdrawal to freezing the sample, which was defined as the pre-processing time (PPT), and finally the sample storage time (SST), which was defined as the time from freezing the sample until arrival at lifespin GmbH, where the serum was prepared for NMR analysis and measured. The major differences in the pre-analytical handling of samples among the cohorts were summarized in Table 3. Blood samples from the PATH biobank were all pre-processed according to the same protocol. Briefly, blood was collected before breast surgery into a 7.5 mL tube with fibrinogen activator (S-Monovette® Serum Gel CAT, Sarstedt). The tube was inverted 2 times to guarantee mixing of the blood with the coagulation factor. The time of blood withdrawal was documented and the tube was stored at 4 °C for at least 30 min and up to 8 h. Then, the blood was centrifuged (10 min, 1,500 g, 4 °C or 20 °C). The serum was then transferred into a 1.5 mL cryovial (Nunc), shock-frozen in liquid nitrogen and stored in the gas phase of liquid nitrogen. Samples were shipped on dry ice for NMR measurement without further thawing.

**TABLE 3:** Pre-analytical differences across sample cohorts.

Sample cohort	Blood collection tube	Volume (ml)	PCT		Centrifugation conditions			max PPT		max SST	Number of freeze-thaw cycles
			t1 (h)	Temp (°C)	Force (g)	Temp (°C)	time (min)	t2 (h)	Temp (°C)	t3 (years)	
URO-IBK pre2012	Serum Gel CAT	4.7	4	20	2061	20	6	8	20	18	2-5
URO-IBK 2012+		4.7	4	20	2061	20	6	48	4 or 20	9	2-5
URO-IBK 2023+		4.7	4	20	2061	20	6	48	4 or 20	2	2-3
URO-PRO	Serum CAT	4.9	1	20	2000	20	10	1.2	20	2	1
Center A	Serum Gel CAT	7.5	8	4	1500	4 or 20	10	8		17	1
Center B		7.5	8	4	1500	4 or 20	10	8		15	1
Center C		7.5	8	4	1500	4 or 20	10	8		15	1
Center D		7.5	8	4	1500	4 or 20	10	8		15	1
Center E		7.5	8	4	1500	4 or 20	10	8		16	1
Center F		7.5	8	4	1500	4 or 20	10	8		16	1
Center G		7.5	8	4	1500	4 or 20	10	8		14	1

Abbr: PCT, pre-centrifugation time; PPT, pre-processing time; SST, sample storage time.

URO-IBK biobank serum samples were collected into 4.7 mL tubes with fibrinogen activator (S-Monovette® Serum Gel CAT, Sarstedt). After withdrawal, the tube was inverted several times and stored at room temperature for up to 8 h until centrifugation (6 min, 2,061 g, 20 °C). Before 2012, the serum samples (URO-IBK pre2012) were pre-processed in the laboratory at the Department of Urology and frozen at −80 °C immediately after the determination of PSA and aliquoting. Samples that were collected after 2012 (URO-IBK 2012+ and URO-IBK 2023+) were stored at 4 °C overnight after centrifugation and then shipped to another lab for routine blood analysis. In case blood was withdrawn during the weekends, the tubes were centrifuged and frozen at −20 °C. These samples were re-thawed after the weekend and shipped to another lab for PSA measurement. After routine analysis, the left-over serum samples were transferred to the URO-IBK biobank, where the serum was pipetted into a 2 mL cryovial (Simport) and frozen at −80 °C. Time from blood withdrawal to freezing was up to 48 h. For shipment on dry ice, the samples were again thawed at 37 °C for 30–60 min depending on the volume, aliquoted, and re-frozen at −20 °C until shipment.

The URO-PRO samples were collected, pre-processed, and stored independently from the URO-IBK biobank samples following a protocol considering the recommendations of the ISO 23118:2021 standard. Briefly, blood was withdrawn into a 4.9 mL tube with fibrinogen activator (S-Monovette® Serum CAT, Sarstedt) up to 24 h before men underwent prostate biopsy due to a suspicion for PCa. The tube was inverted 2 times and stored at room temperature for at least 30 min in an upright position. Then samples were centrifuged (10 min, 2000 g, 20 °C), aliquoted (2 × 500 µL) into 2 mL cryovials (Simport) and frozen at −80 °C within 1.2 h after blood withdrawal. Samples were shipped on dry ice for NMR measurement without further thawing.

### NMR measurement and data analysis

Targeted metabolic profiling was performed at lifespin GmbH (Regensburg, Germany) by NMR spectroscopy. Frozen serum samples were thawed for 0.5–1 h at room temperature. Only some single cases were thawed for about 3 h at room temperature reliant on the daily routine workload. Then, 350 µL of serum were mixed with 350 µL of aqueous buffer containing 0.1 g/L NaN_3_, 0.067 mol/L Na_2_HPO_4_, 0.033 mol/L NaH_2_PO_4_ (pH = 7.15 ± 0.05), 5% deuterium oxide (D_2_O) as field-lock substance, and 6 mM pyrazine that was used as an internal standard for quantification. From this mixture, 600 µL were transferred into a 5 mm Bruker NMR tube and sealed with barcode-labelled caps. The final NMR samples were stored at 4 °C for a maximum of 24 h until measurement. NMR measurement was performed using a Bruker AVANCE NEO 600 MHz spectrometer using 1D 1H noesygppr1d_d20, NS = 16, T = 310K as measuring method. Measuring time per sample was 6.5 min. All measured spectra passed the routine quality control and were released for data analysis. The spectra obtained were Fourier transformed using TopSpin software (version 4.1.1 and 4.2.0, Bruker Biospin, Germany). All spectra were automatically phased and subjected to baseline correction. Subsequently, the spectra were analyzed using the proprietary lifespin Profiler software (version 1.4_Blood) and lipoprotein profiler software (version 1.2.3_A) to generate a quantitative list for all metabolites (Supplementary Table S1). In brief, the metabolites were measured in pure form and individual reference files were generated for each metabolite, which were then reproduced in the more complex blood spectra using fitting algorithms for both small metabolites and albumin. When quantifying lipoproteins, the corresponding lipid signal was replicated using several individual fitting algorithms and the concentrations were inferred using a particle model. The concentration of the metabolites was given in mmol/L, the concentration of the lipoprotein parameters in mg/dL with the mean particle diameter being given in nm, and the concentration of lipoprotein particles in nmol/L.

### Statistics

All statistical analyses and model building was performed using R statistical software (version 4.0.2; R Core Team, 2025). For any model building (PCA, PLS-DA), the nearZeroVar function in R was used to remove zero variance predictors (i.e., metabolites, which have very few unique values relative to the number of samples) to improve stability and interpretability of any subsequent models. For the multivariate analysis, a Principle Component Analysis (PCA) and a Partial Least Squares Discriminant Analysis (PLS-DA) was applied. The PCA is an unsupervised statistical method used for dimensionality reduction. The original data set is transformed into a smaller set of uncorrelated variables, the so-called principal components. These capture the maximum possible variance within the first few components and as such enable the visualization of inherent data structures. The PLS-DA, a supervised classification method, is used for the identification of variables with maximal discrimination between the classes. Hereby, the relationship between the dataset X (measured data points) and its labels Y (categorial group classifications) is considered and a set of components is established which maximize the covariance between X and Y. Statistical significance was determined using the Wilcoxon-Mann-Whitney-U-Test and FDR-corrected for multiple testing. *P*-values were translated into * notation as follows: *** indicates p ≤ 0.001, ** indicates p ≤ 0.01, and * indicates p ≤ 0.05. Effect sizes were indicated by the absolute value of Cohen’s d, which was calculated by dividing the difference of the means of the respective groups by their pooled standard deviation. Considering absolute Cohen’s d values, effects were interpreted as small with a Cohen`s d value <0.5, between 0.5 and 0.8 as medium, and >0.8 as large. A negative Cohen’s d value implies that the mean of the second group is higher than the mean of the first group. Whiskers in box plots were plotted with the furthest data point that is still within 1.5 times the interquartile range (IQR) from the edges of the box.

## Results

### Metabolite concentrations in relation to sample storage time (SST) and center differences

**FIGURE 2:**
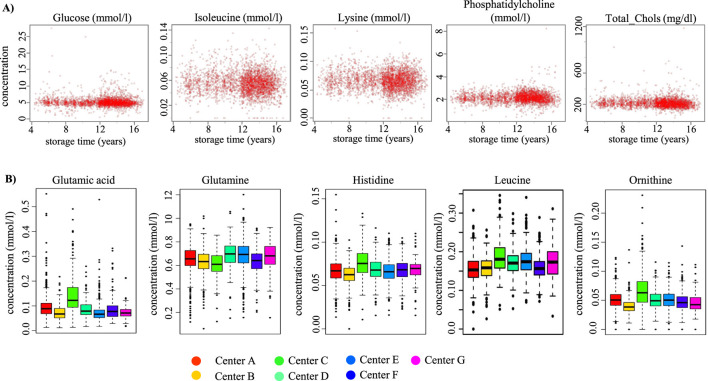
Comparison of metabolite concentrations in relation to SST and among the 7 PATH centers. **(A)** Scatter plots showing metabolite serum concentrations of all PATH biobank samples in relation to the SST. Each dot represents an individual serum sample. Concentrations were given in mmol/L or mg/dL (total_chols, total cholesterol). **(B)** Comparison of mean metabolite concentrations across serum samples of the different PATH centers (A–G) expressed as box plots with whiskers.

We first addressed the question whether the SST had an influence on metabolite concentrations. To this end, we compared PATH biobank female serum samples from BCa patients. These samples displayed different SSTs with equal PPTs (Table 2) since the samples were pre-processed in 7 different centers using the same pre-analytical protocol from blood withdrawal until NMR analysis. In addition, these samples were all stored at the PATH biobank under the same conditions. Overall, we observed a good concordance of the samples with different SSTs. Most metabolites such as glucose, isoleucine, lysine, phosphatidylcholine, and total cholesterol were not or only marginally affected by the SST (Figure 2A). Of note, when considering the individual locations separately, differences in mean concentrations were found for some individual metabolites, including histidine, glutamic acid, glutamine, leucine, and ornithine (Figure 2B). In particular, we observed higher mean concentrations of glutamic acid, histidine, leucine, and ornithine and lower levels of glutamine in one of the centers (PATH C) compared to the other centers.

### Influence of the pre-centrifugation time (PCT) on metabolite concentrations

The sample pre-processing can be distinguished into two working steps: the time between blood withdrawal and centrifugation of the sample, which was named PCT, and the other between centrifugation and freezing of the sample (Figure 1). We tested the influence of the PCT on metabolite concentrations. To this end, we took seven fresh blood samples from healthy volunteers and subdivided each of them into two separate test tubes, which were allowed to stand in an upright position for 30 min and 6 h, respectively. Then, the tubes were centrifuged and the serum metabolites measured by NMR spectroscopy. We primarily focused on four metabolites and their respective ratios (glucose:lactate, glutamic acid:glutamine), which were significantly influenced by the PCT in our study and which have been previously considered as potential quality control markers for metabolomics (Malm et al., 2016; Valo et al., 2022). The ratio of glucose: lactate in the samples with a PCT of 30 min was >1.5 whereas the ratio in the samples with a PCT of 6 h was <1.5. Similarly, with respect to the ratio of glutamine:glutamic acid, optimal group separation was achieved for a ratio of 5.4, i.e., the ratio in the samples with a PCT of 30 min was >5.4 whereas the ratio in the samples with a PCT of 6 h was <5.4.

**FIGURE 3:**
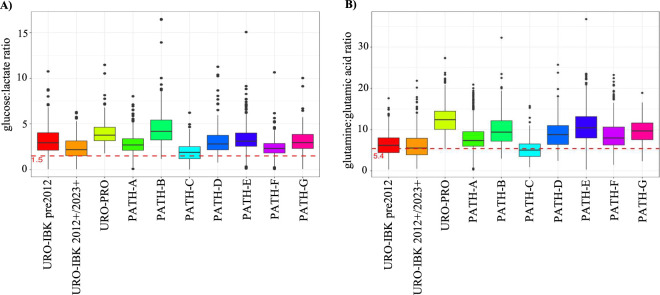
Influence of the PCT on the ratios of glucose:lactate **(A)** and glutamine:glutamic acid **(B)** in the different cohorts. The dotted red lines indicate the thresholds for pre-analytically critical values with respect to the PCT, which were defined with <1.5 for glucose:lactate and <5.4 for glutamine:glutamic acid ratios, respectively. Values per cohort were expressed as box plots with whiskers.

Based on these findings, we further evaluated these four metabolites for their usefulness as quality control markers using a threshold of glucose:lactate <1.5 and glutamine:glutamic acid <5.4 as preanalytically conspicuous with respect to sample quality. As summarized in Figure 3A, serum samples from the URO-IBK 2012+/2023+ and the PATH-C cohorts exhibited the lowest glucose:lactate ratio with some samples even featuring a glucose:lactate ratio below the threshold of 1.5. The highest glucose:lactate ratio, on the other hand, was found in samples from the URO-PRO cohort and the PATH-B cohort. Similarly, the URO-PRO cohort exhibited the highest glutamine:glutamic acid ratio, whereas the lowest values were revealed in the URO-IBK 2012+/2023+ as well as in samples collected at the PATH-C cohort (Figure 3B). However, it has to be emphasized that the threshold of 1.5 and 5.4 was formed based on 6 h of PCT, and the study protocols allowed for up to 8 h of PCT. Hence, it is only sensible to detect samples below the thresholds. All in all, these data suggest that the glucose:lactate as well as the glutamine:glutamic acid ratios display deviations in the PCT during the pre-analytical processing of the samples.

### Influence of the pre-processing time (PPT) on metabolite concentrations

**FIGURE 4:**
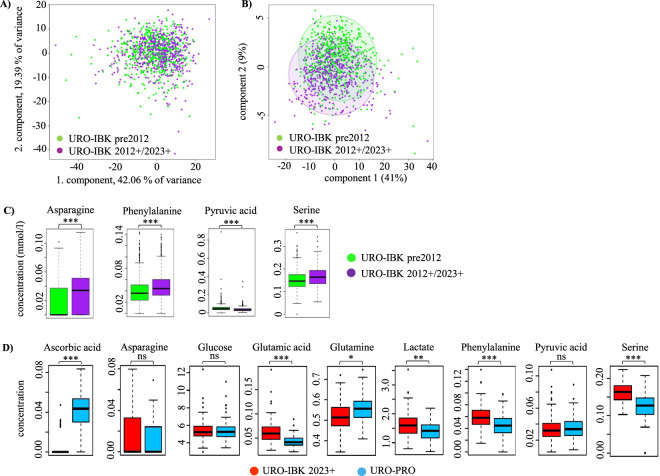
Influence of the PPT on metabolite concentrations. Principle component analysis (PCA) **(A)** and PLS-DA analysis **(B)** was performed to evaluate differences in metabolites between two cohorts with different SST and PPTs. **(C)** Metabolite concentrations in samples from cohorts with different SST, and PPT. **(D)** Metabolite concentrations in patient-matched serum samples with equal SST but different PCT and PPT. Metabolite concentrations were given in mmol/L and expressed as box plots with whiskers. ***p < 0.001, **p < 0.01, *p < 0.05, ns *p* > 0.05.

We next looked at the impact of the PPT on metabolite concentrations. For this, we used the URO-IBK pre2012 and URO-IBK 2012+/2023+ cohorts, which differ in their PPTs due to a significant change in the process workflow, which resulted in a significant extension of the PPT from 8 to 48 h (Table 2). This change was necessary because the routine diagnosis of PSA was transferred from the laboratory of the Department of Urology to the central laboratory of the general hospital in Innsbruck. As a consequence, the blood samples were not brought to the laboratory for measurement directly after withdrawal as in former years, but shipped to another lab for clinical diagnosis instead. From this external lab, the remaining serum was retrieved back after analysis for final storage at −80 °C in the URO-IBK biobank. We therefore separated the serum samples into the two different cohorts URO-IBK pre2012 and URO-IBK 2012+, respectively. In addition, we looked at short term stored serum samples (URO-IBK 2023+), which were collected with the same pre-processing protocol as URO-IBK 2012+ samples and which were therefore grouped together. As shown in Figure 4A, the samples showed an overall good concordance for the majority of the metabolites across the three cohorts independent of the SST. A PLS-DA analysis also revealed an overall strong overlap of URO-IBK pre2012 and URO-IBK 2012+/2023+ samples, however, still some group separation was observed within the PLS-DA, indicating that a few metabolites differ between the two cohorts (Figure 4B).

To further investigate the influence of the PPT on metabolite concentrations, we selected a few metabolites which exhibited the highest significant difference and the largest effect size (Cohen’s d > 0.3) between the two cohorts, namely, asparagine (Cohen’s d −0.55), phenylalanine (Cohen’s d −0.38), pyruvic acid (Cohen’s d 0.42), and serine (Cohen’s d −0.40), and analyzed them in more detail. As shown in Figure 4C, asparagine, phenylalanine, and serine concentrations were significantly lower (*p* < 0.001) in URO-IBK pre2012 compared to URO-IBK 2012+/2023+ samples, whereas pyruvic acid concentrations were significantly higher (*p* < 0.001). These data show that asparagine, phenylalanine, pyruvic acid, and serine concentrations reflect differences in the PPT among samples. Of note, many samples of the URO-IBK pre2012 cohort exhibited asparagine concentrations below the detection limit and which were therefore represented by a zero value.

To further validate our observations on the impact of PCT and PPT on metabolite concentrations, we next looked at short-term stored patient-matched serum samples from the URO-IBK 2023+ and the URO-PRO cohort. These two cohorts consist of serum samples which were withdrawn from the same patients into two different tubes, which were further pre-processed with different protocols (Table 2). In fact, the URO-IBK 2023+ samples exhibited much longer standing times (PCT and PPT) than the samples from the URO-PRO cohort but the same SST, thereby allowing a direct comparison of metabolite concentrations in samples from one patient. Corresponding to our previous results, phenylalanine, and serine were higher in the URO-IBK 2023+ compared to the URO-PRO cohort, indicating that the concentrations of these metabolites are significantly affected by longer PCT and PPT (Figure 4D). In addition, glutamic acid and lactate were significantly higher in the URO-IBK 2023+ compared to the URO-PRO cohort, whereas ascorbic acid and glutamine were significantly lower in the URO-IBK 2023+ samples with longer PCT and PPT. No significant effect was found for asparagine, glucose and pyruvic acid.

### Impact of sample texture on metabolite concentrations

**FIGURE 5:**
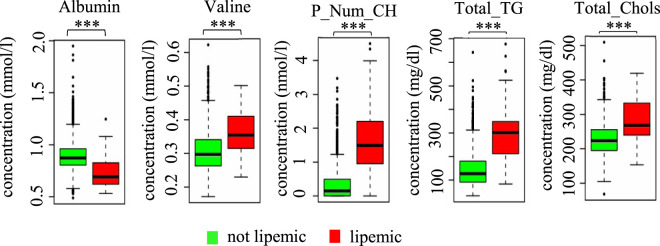
Impact of sample texture on metabolite concentrations. Serum samples were subdivided into lipemic and not lipemic based upon clouding and appearance of a white lipid film on the surface of the sample upon sample preparation for NMR analysis. Albumin and valine were given in mmol/L, particle number of chylomicrons (P_Num_CH) in nmol/L, total triglycerides (Total_TG), and total cholesterol (Total_Chols) in mg/dL. Concentrations were expressed as box plots with whiskers. ***p < 0.001.

Contaminations with red blood cells are known to interfere with accurate metabolite quantification (Denihan et al., 2015; Searfoss et al., 2022). Therefore, hemolytic blood samples were primarily sorted out by visual examination and not included into NMR spectroscopy measurements. Lipemic serum samples, by contrast, were examined upon their usefulness for NMR-based metabolomics. We analysed serum samples from long-term stored URO-IBK cohorts (pre2012, 2012+), which were defined as lipemic when they exhibited large clouding and a separation of a white lipid film on the sample surface after sample preparation for NMR analysis. As shown in Figure 5, we found for instance significant differences in the concentrations of albumin and valine, particle number of chylomicrons (P_Num_CH), total triglycerides (Total_TG), and total cholesterol (Total_Chols) in lipemic compared to non-lipemic serum samples (*p* < 0.001***). These data suggest that the sample texture has a significant impact on the concentrations of selected metabolites and that care must be taken with the analysis of lipemic samples.

### Impact of potential pre-analytical and analytical contaminations

**FIGURE 6:**
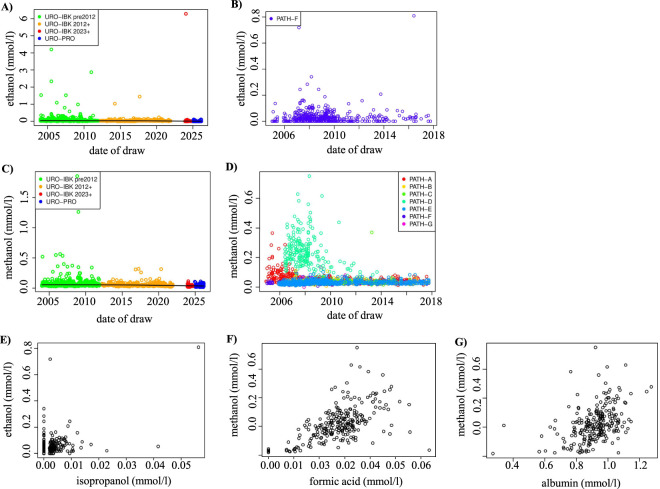
Influence of ethanol and methanol levels on metabolite analysis in the different cohorts. Ethanol **(A,B)** and methanol **(C,D)** levels were compared across the different cohorts. Scatter plots were used to show the correlation of ethanol to isopropanol in the PATH-F cohort **(E)** and of methanol to formic acid and albumin in the PATH-D cohort **(F,G)**.

The NMR spectroscopy protocol currently used enables the quantification of 250 different metabolites in one single measurement (Supplementary Table S1) at concentrations of 1 μmol/L or higher. Based on this high sensitivity of the method, accurate interpretation of the spectra for metabolite quantification may be hampered by contaminations with residues from pre-analytical chemicals or disinfection solvents. In some cohorts, we in fact observed unusually high levels of ethanol (Figure 6A) and methanol (Figure 6C), respectively. Particularly high levels of ethanol were observed in samples from the PATH-F cohort (Figure 6B), whereas methanol levels were explicitly high in PATH-D sera (Figure 6D), suggesting probable pre-analytical contaminations.

Based on these findings, we considered the correlation between ethanol and methanol and all other metabolites for the PATH-F and the PATH-D cohorts, respectively. To this end, we applied distance, Spearman, and Pearson correlations to detect potential relationships. All metabolites with a distance correlation coefficient >0.3 were considered to potentially detect also small effects. As summarized in Table 4, isopropanol was identified in the PATH-F cohort with a correlation to ethanol of greater than 0.3. In the PATH-D cohort, 8 metabolites were found with a correlation to methanol greater than 0.3. In particular formic acid and albumin showed the strongest effect. However, the correlation coefficient of all three metabolites remained below a value of 0.6, indicating that ethanol and methanol do not affect the majority of the analyzed metabolites and only have a moderate effect on these three metabolites (Figures 6E–G).

**TABLE 4:** Pre-analytical differences across sample cohorts. Distance correlation, Spearman and Pearson correlation were calculated. Only metabolites with a distance correlation >0.3 were considered and to accompany the distance correlation, a Monte-Carlo permutation p-value was determined based on 999 random permutations.

Center	Metabolite	Distance correlation	Permutation p-value (R = 999)	Spearman	Pearson
PATH-F					
	Isopropanol	0.476	0.110	0.482	0.510
PATH-D					
	Formic acid	0.582	0.001	0.574	0.561
	Albumin	0.439	0.001	0.454	0.423
	Alanine	0.348	0.001	0.376	0.293
	Acetoacetic acid	0.347	0.001	−0.392	−0.212
	3-Hydroxybutyric acid	0.314	0.001	−0.351	−0.085
	P_Num_HDL4	0.308	0.001	0.325	0.306
	ApoA2_HDL4	0.308	0.001	0.323	0.305
	ApoA1_HDL4	0.303	0.001	0.315	0.299

Abbr: P_Num, Particle Number; ApoA2, Apolipoprotein A2; ApoA1, Apolipoprotein A1; HDL, high density lipoprotein.

## Discussion

Metabolomics has emerged as an important tool to investigate metabolic changes in various diseases and to establish novel biomarkers for diagnosis. Most studies use biobanked samples, which are usually stored over several years under different conditions and which use different protocols for pre-processing. The comparison of metabolomics data and the usefulness of such long-term stored serum samples for metabolomics therefore has been largely debated until now. In this study, we looked at 3,262 serum samples from females with BCa, which were stored in the gas phase of liquid nitrogen for up to 17 years, and which followed one equal pre-processing protocol. Our results generally demonstrated that the majority of metabolites were largely stable and showed a good concordance among the samples. In fact, there were only a few metabolites, which varied across the different centers, including glucose, lactate, glutamic acid, glutamine, and ornithine. Of note, all of these samples were stored in liquid nitrogen, which should be favored over a storage at −80 °C in particular for metabolomics according to previous reports (Emwas et al., 2025) because of efficient and rapid quenching of endogenous enzymes in liquid nitrogen, which further prevents metabolic activity and degradation of metabolites (Gonzalez-Dominguez et al., 2020).

Nevertheless, there was also a good concordance of most of the metabolites in the 1,396 male serum samples, which were stored at −80 °C, though a direct comparison of the two storage conditions cannot be made since these samples were from men. Moreover, these serum samples were not only derived from PCa patients (n = 623) but also from healthy men as well as from patients with benign prostate diseases such as benign prostatic hyperplasia and prostatitis (n = 773). The impact of biological differences between women and men, including differences in the hormonal status and body composition are known to cause variations in metabolite concentrations (Emwas et al., 2025). Due to major differences in the pre-analytical workflow, the samples from URO-IBK were also not used to investigate the impact of SST on metabolite concentrations but to evaluate the influence of delays in the PPT instead. Previous studies have shown that the pre-processing conditions, in particular the PPT and the respective temperatures at each work step, have a large impact on the sample quality (Salciccia et al., 2021). Our study revealed that despite varying pre-processing protocols, there was an overall good conformity across the cohorts with regard to metabolite concentrations, suggesting that most of the metabolites are stable enough for metabolomic investigations. These findings are in concordance with other previous studies showing reproducible and stable metabolomics data across several different cohorts with different pre-processing protocols (Ward et al., 2010; La Frano et al., 2018).

Importantly, however, we also identified a few individual metabolites, which were significantly influenced by the PCT and PPT. Among the most significantly PCT and PPT-sensitive metabolites in this study were ascorbic acid, asparagine, glucose, lactate, glutamic acid, glutamine, phenylalanine, pyruvic acid, and serine. Rapid degradation of ascorbic acid, as we observed it in this study, has also been previously reported by Karlsen and colleagues, who suggested to centrifuge and freeze the samples as immediately as possible after withdrawal (Karlsen et al., 2007). Phenylalanine and glutamic acid were previously shown to significantly increase by longer PPTs in a study performing mass spectrometric analysis of metabolites (An et al., 2021). Another study found increased concentrations of phenylalanine and serine in samples with a pre-storage handling of 36 h (Anton et al., 2015). In addition, Thachil and coworkers reported on depletion of glucose and pyruvic acid and accumulation of lactate, most likely due to ongoing cellular metabolism prior to centrifugation (Thachil et al., 2024). Lactate, in particular, accounts as one of the most sensitive metabolites, whose serum concentrations may change even within 30 min after blood withdrawal (Seymour et al., 2011). This increase in lactate levels due to delays in the PCT most likely results from glycolysis in erythrocytes (Debik et al., 2022a; Debik et al., 2022b) or residual enzyme activity from damaged cells, which increases upon longer preparation times (Santos Ferreira et al., 2019). In this study, we showed that serum samples from our IBK-PRO cohort, which were pre-processed within 1.2 h, exhibited the highest glucose:lactate ratio compared to URO-IBK 2012+/2023+ samples, for which the PPT was up to 48 h, suggesting the glucose:lactate ratio as an indicator for a delay in the PCT or PPT. Similarly, the delayed PPT in the URO-IBK 2012+/2023+ cohort was associated with a lower ratio of glutamine:glutamic acid compared to the URO-PRO cohort. Overall, our data indicate that the time between blood withdrawal and freezing of the sample is an intriguing factor with regard to metabolomics and should be kept as short as possible in particular for PPT-sensitive metabolites, including ascorbic acid, asparagine, glucose, lactate, glutamic acid, glutamine, phenylalanine, pyruvic acid, and serine. Moreover, the ratios of glucose:lactate and glutamine:glutamic acid may be used to assess sample quality in case of PCT delays of more than 6 h.

Since the limitations of retrospective metabolomic studies with regard to protocol variations are a matter of common knowledge, it is not surprising that the use of standardized protocols has been highly recommended in the past (Emwas et al., 2025; Powers et al., 2024). Recently, Emwas and colleagues published recommendations for the sample pre-processing and storage for metabolomics analysis emphasizing the strong requirement for freezing the samples as fast as possible within 1 hour (Emwas et al., 2025). Since 2021, there is an international pre-analytical sample preparation standard (ISO 23118:2021) available, which defines several points within the pre-analytical and analytical handling that should be strongly considered. This standard covers all possible considerations at the different work steps, including a strict protocol for the PPT and temperature. In addition, this standard recommends to consider several other important issues which influence the metabolomics profile such as the time of blood withdrawal for instance. Previous studies have outlined that the circadian rhythm of the sample donors, their nutritional status, physical activity, medication, sex, and age may result in unanticipated variance of metabolite concentrations (Stringer et al., 2015). Glutamine and lactate, for instance, were reported to be age-dependent (van den Akker et al., 2020). We considered the recommendations of the ISO 23118:2021 standard in the URO-PRO cohort, which started in 2023. In this cohort, we not only followed the respective pre-processing protocol but also considered patient-derived confounders like weight, body mass index, health status, diet, and medication using a questionnaire for patient selection. The collection of the samples for the other cohorts started before the publication of the standard. Besides, it should be noted that despite the undisputed importance of standardized procedures, it must be considered that taking into account all the recommended parameters during the pre-analytical process may be challenging, in particular within clinical routine and may require additional personnel and also budget. Hence, to save costs and to include sample collection into clinical routine management, protocols may be slightly adapted to the specific requirements of the research interests and the administrative feasibilities of the respective center. Like that, broader time frames for the PPT from 30 min to 8 h or even up to 48 h may cause variations in metabolite concentrations.

When using metabolomic technologies such as mass spectrometry or NMR, it must be additionally taken into account that in contrast to standard clinical chemistry techniques, hundreds to thousands of metabolites can be detected in one biological sample in a single measurement at very low concentrations (Sun and Xia, 2024). Though this is of course desirable on the one hand, care must be taken with the sample texture and analytical interference from solvent or buffer residues on the other. The most common analytical interference in clinical laboratory analysis includes hemolysis and lipemia. Contamination with red blood cells from hemolysis is known to interfere with a variety of laboratory assays, including metabolomics (Stringer et al., 2015). In this study, we excluded hemolytic samples from the analysis but we investigated whether lipemic samples could be used for NMR analysis. The concentrations of valine, and various lipid metabolites, including the particle number of chylomicrons, total cholesterol and total triglycerides, were significantly higher in lipemic compared to non-lipemic samples, and significantly lower for albumin. A previous study recently showed that lipemic samples accumulate large numbers of lipoproteins, which are rich in triglycerides and which contribute to sample turbidity consequently influencing metabolomics (Prendes et al., 2023). Lipemic samples should therefore be carefully interpreted with regard to metabolite concentrations. In this respect, though not addressed in this study, not only the PCT but also the sort of blood collection tubes, the pre-centrifugation temperature, and the centrifugation conditions–particularly the centrifugation force–and the number of freeze- -cycles may influence metabolite concentrations (Ghini et al., 2019; Gonzalez-Dominguez et al., 2020). A recent study observed that there were only minimal to no changes in lipid metabolite concentrations when samples were frozen and re-thawed for up to 4 times (Loo et al., 2020). Similarly, Anton et al. found only minimal changes in some amino acids with higher numbers of freeze-thaw cycles (Anton et al., 2015).

A recent study compared the metabolomics and lipoprotein profiles in blood samples collected using different collection tubes, including citrate plasma, EDTA plasma and serum, and found significant changes in several metabolites among the different blood matrices (Vignoli et al., 2022). Emwas et al. also pointed towards the possibility that anti-clotting agents such as EDTA and heparin may interfere with metabolite spectra (Emwas et al., 2025). In our study, serum monovettes with a gel to improve blood coagulation were used to collect samples in the URO-IBK and the PATH serum samples, respectively, whereas tubes without gel were used to collect the samples for the URO-PRO cohort. The latter in fact exhibited the highest sample quality, indicating that a clotting time between 30 and 60 min and the use of serum monovettes without a gel are recommended to guarantee a good quality for metabolomics. A recent study suggested that tube-dependent changes might be minimized by using regression analysis, however, in practical work, the use of the same type of tube for multicenter studies is highly recommended (Nagana Gowda and Raftery, 2023).

Unanticipated variance of metabolite concentrations may also result from solvent or buffer residues during sample processing or NMR measurement. In the present study, some samples contained exceptionally high concentrations of ethanol and methanol, respectively. Ethanol produces a strong signal in NMR spectroscopy, which may interfere with the detection and quantification of other metabolites. High levels of ethanol may derive from skin disinfectants or routinely used sterilization buffers in the lab during pre-processing and was shown to easily diffuse into samples via air transmission (van der Sar et al., 2015). These authors therefore recommended to avoid the use of ethanol for sample preparation and to work in a well-ventilated atmosphere. High levels of ethanol in serum, on the other hand, may also trace back to recent alcohol consumption of the donor.

Summarizing our data, we conclude that even long-term stored serum samples collected at different centers with varying protocols can be used for metabolomics when the interpretation of individual metabolites takes limitations and confounding factors due to variations in the pre-processing into account. Furthermore, for valid comparison of published metabolomics data it is necessary to document all information about the pre-analytical conditions. As a recent review revealed, unfortunately, this information is lacking in two-thirds of published studies (Emwas et al., 2025), rendering a comparison of metabolomics data still difficult. We further strongly recommend the use of internal quality control markers based on specific metabolites in the clinical diagnosis. In any case, care must be taken in the handling and pre-processing of the samples due to the high susceptibility of some metabolites to small process changes and therefore–in particular for newly started projects - the strict adherence to a standardized protocol is essential.

## Data Availability

The raw data supporting the conclusions of this article will be made available by the authors, without undue reservation.
