# Supplementary material for: Pitfalls of onco-metabolomics: impact of sample integrity on metabolomic investigations in more than 4500 human serum samples from ten different cohorts
Source: Front Mol Biosci. 2026 Mar 31;13:1765747. doi: 10.3389/fmolb.2026.1765747 (PMC13076171; doi:10.3389/fmolb.2026.1765747)
Supplement: Supplementary file 1 [file Table1.docx]

| **Supplementary Table S1: List of all metabolized analyzed.** |
| --- |
| 1-Methylhistidine |
| 1,2-Propanediol |
| 2-Aminobutyric acid |
| 2-Hydroxyisobutyric acid |
| 2-Methyl-1,3-propanediol |
| 2,3-Butanediol |
| 3-Hydroxybutyric acid |
| 3-Methylhistidine |
| Acetic acid |
| Acetoacetic acid |
| Acetone |
| Adenine |
| Alanine |
| Albumin |
| Arginine |
| Ascorbic acid |
| Asparagine |
| Aspartic acid |
| beta-Alanine |
| Betaine |
| Carnitine |
| Choline |
| Creatine |
| Creatinine |
| Cystine |
| Dimethylamine |
| Dimethylglycine |
| Dimethylmalonic acid |
| Dimethylsulfone |
| Ethanol |
| Ethanolamine |
| Formic acid |
| Fumaric acid |
| Glucose |
| Glucuronic acid |
| Glutamic acid |
| Glutamine |
| Glycerol |
| Glycine |
| Glycoprotein acetyls A |
| Glycoprotein acetyls B |
| Histidine |
| Hypoxanthine |
| Isoleucine |
| Isopropanol |
| Lactate |
| Leucine |
| Lysine |
| Maleic acid |
| Mannitol |
| Mannose |
| Methanol |
| Methionine |
| Methylamine |
| Methylmalonic acid |
| myo-Inositol |
| Ornithine |
| Phenylalanine |
| Phosphatidylcholine |
| Proline |
| Propionic acid |
| Pyruvic acid |
| Sarcosine |
| Serine |
| Serotonin |
| Sphingomyeline |
| Succinic acid |
| Threonine |
| Total protein |
| Trimethylamine |
| Tyrosine |
| Urea |
| Valine |
| Total_Chols |
| Total_CE |
| Total_FC |
| Total_PL |
| Total_TG |
| Total_ApoA1 |
| Total_ApoA2 |
| Total_ApoB |
| Total_P_Num |
| non_HDL_P_Num |
| P_Num_HDL4 |
| Chols_HDL4 |
| CE_HDL4 |
| FC_HDL4 |
| PL_HDL4 |
| TG_HDL4 |
| ApoA1_HDL4 |
| ApoA2_HDL4 |
| Mean_Part_Diam_HDL4 |
| P_Num_HDL3 |
| Chols_HDL3 |
| CE_HDL3 |
| FC_HDL3 |
| PL_HDL3 |
| TG_HDL3 |
| ApoA1_HDL3 |
| ApoA2_HDL3 |
| Mean_Part_Diam_HDL3 |
| P_Num_HDL2 |
| Chols_HDL2 |
| CE_HDL2 |
| FC_HDL2 |
| PL_HDL2 |
| TG_HDL2 |
| ApoA1_HDL2 |
| ApoA2_HDL2 |
| Mean_Part_Diam_HDL2 |
| P_Num_HDL1 |
| Chols_HDL1 |
| CE_HDL1 |
| FC_HDL1 |
| PL_HDL1 |
| TG_HDL1 |
| ApoA1_HDL1 |
| ApoA2_HDL1 |
| Mean_Part_Diam_HDL1 |
| P_Num_HDL |
| Chols_HDL |
| CE_HDL |
| FC_HDL |
| PL_HDL |
| TG_HDL |
| ApoA1_HDL |
| ApoA2_HDL |
| Mean_Part_Diam_HDL |
| P_Num_LDL6 |
| Chols_LDL6 |
| CE_LDL6 |
| FC_LDL6 |
| PL_LDL6 |
| TG_LDL6 |
| ApoB_LDL6 |
| Mean_Part_Diam_LDL6 |
| P_Num_LDL5 |
| Chols_LDL5 |
| CE_LDL5 |
| FC_LDL5 |
| PL_LDL5 |
| TG_LDL5 |
| ApoB_LDL5 |
| Mean_Part_Diam_LDL5 |
| P_Num_LDL4 |
| Chols_LDL4 |
| CE_LDL4 |
| FC_LDL4 |
| PL_LDL4 |
| TG_LDL4 |
| ApoB_LDL4 |
| Mean_Part_Diam_LDL4 |
| P_Num_LDL3 |
| Chols_LDL3 |
| CE_LDL3 |
| FC_LDL3 |
| PL_LDL3 |
| TG_LDL3 |
| ApoB_LDL3 |
| P_Num_LDL2 |
| Chols_LDL2 |
| CE_LDL2 |
| FC_LDL2 |
| PL_LDL2 |
| TG_LDL2 |
| ApoB_LDL2 |
| Mean_Part_Diam_LDL2 |
| P_Num_LDL1 |
| Chols_LDL1 |
| CE_LDL1 |
| FC_LDL1 |
| PL_LDL1 |
| TG_LDL1 |
| ApoB_LDL1 |
| Mean_Part_Diam_LDL1 |
| P_Num_LDL |
| Chols_LDL |
| CE_LDL |
| FC_LDL |
| PL_LDL |
| TG_LDL |
| ApoB_LDL |
| Mean_Part_Diam_LDL |
| P_Num_IDL |
| Chols_IDL |
| CE_IDL |
| FC_IDL |
| PL_IDL |
| TG_IDL |
| ApoB_IDL |
| P_Num_VLDL5 |
| Chols_VLDL5 |
| CE_VLDL5 |
| FC_VLDL5 |
| PL_VLDL5 |
| TG_VLDL5 |
| ApoB_VLDL5 |
| Mean_Part_Diam_VLDL5 |
| P_Num_VLDL4 |
| Chols_VLDL4 |
| CE_VLDL4 |
| FC_VLDL4 |
| PL_VLDL4 |
| TG_VLDL4 |
| ApoB_VLDL4 |
| Mean_Part_Diam_VLDL4 |
| P_Num_VLDL3 |
| Chols_VLDL3 |
| CE_VLDL3 |
| FC_VLDL3 |
| PL_VLDL3 |
| TG_VLDL3 |
| ApoB_VLDL3 |
| Mean_Part_Diam_VLDL3 |
| P_Num_VLDL2 |
| Chols_VLDL2 |
| CE_VLDL2 |
| FC_VLDL2 |
| PL_VLDL2 |
| TG_VLDL2 |
| ApoB_VLDL2 |
| Mean_Part_Diam_VLDL2 |
| P_Num_VLDL1 |
| Chols_VLDL1 |
| CE_VLDL1 |
| FC_VLDL1 |
| PL_VLDL1 |
| TG_VLDL1 |
| ApoB_VLDL1 |
| Mean_Part_Diam_VLDL1 |
| P_Num_VLDL |
| Chols_VLDL |
| CE_VLDL |
| FC_VLDL |
| PL_VLDL |
| TG_VLDL |
| ApoB_VLDL |
| Mean_Part_Diam_VLDL |
| P_Num_CH |
| Chols_CH |
| CE_CH |
| FC_CH |
| PL_CH |
| TG_CH |
| ApoB_CH |
| Mean_Part_Diam_CH |
| Total_ApoB%Total_ApoA1 |
| Chols_LDL%Chols_HDL |
| FC_HDL%Chols_HDL |
| FC_LDL%Chols_LDL |
